# Effectiveness of the Japanese standard family psychoeducation on the mental health of caregivers of young adults with schizophrenia: a randomised controlled trial

**DOI:** 10.1186/s12888-019-2252-y

**Published:** 2019-09-02

**Authors:** Nao Shiraishi, Norio Watanabe, Fujika Katsuki, Hajime Sakaguchi, Tatsuo Akechi

**Affiliations:** 1 Department of Psychiatry and Cognitive-Behavioral Medicine, Nagoya City University Graduate School of Medical Sciences, 1 Kawasumi, Mizuho-cho, Mizuho-ku, Nagoya, 467-8601 Japan; 2 Departments of Health Promotion and Human Behavior and of Clinical Epidemiology, Kyoto University Graduate School of Medicine/School of Public Health, Yoshida Konoe-cho, Sakyo-ku, Kyoto, 606-8501 Japan; 3 Department of Psychiatric and Mental Health Nursing, Nagoya City University School of Nursing, 1 Kawasumi, Mizuho-cho, Mizuho-ku, Nagoya, 467-8601 Japan; 4 Midorino Kaze Minamichita Hospital, Magohazama-86 Toyooka, Minamichita, Aichi, Chita District 470-3411 Japan

**Keywords:** Care burden, Psychotic disorders, Young adulthood, Family intervention, Multicentre study

## Abstract

**Background:**

This study examined the effects of the standard model of family psychoeducation (SM-FPE) in Japan on the mental health of relatives who care for young patients with a psychotic disorder.

**Methods:**

Stratified by recent-onset/chronic psychosis, 74 caregivers of outpatients aged 30.1 years (mean) were randomly assigned to receive TAU (treatment as usual) alone or TAU plus SM-FPE. All outcomes were measured at baseline, at the end of the intervention (10 weeks), and 1 month post-intervention (14 weeks). The primary outcome was the trait anxiety of caregivers at 14 weeks. Secondary outcomes included caregivers’ state anxiety, psychological distress, care burden, and expressed emotion. Integrating these secondary outcomes, a conceptual framework of caregivers’ health state was assessed via structural equation modelling.

**Results:**

Compared with TAU alone, SM-FPE plus TAU did not significantly improve all caregivers’ individual outcomes. Direct effects of the intervention were observed in the caregivers of chronic patients as significant improvements of their overall mental health state at 10 weeks, which indirectly continued until 14 weeks. However, such intervention effects were not observed in the caregivers of recent-onset patients.

**Conclusions:**

The lack of effectiveness in the recent-onset stage suggests that the usefulness of the SM-FPE needs to be corroborated by further research.

**Trial registration:**

The study protocol was retrospectively registered at ClinicalTrials.gov (registration number: NCT01731977; date of registration: 22 November 2012).

## Background

Psychosis, principally including schizophrenia, is a severe pathological condition that commonly develops in one’s twenties and often leads to a chronic course. Individuals with schizophrenia may experience years of disability, which also imposes a considerable burden on their family caregivers [1]. The burden of care is defined by the disorder’s impact and consequences on caregivers and has objective and subjective components. The subjective components relate to the perception of situations related to care, which can cause a psychological burden [2–4]. Relatives caring for patients with early stages of psychosis tend to suffer from distress, comparable to those caring for chronic patients [5–7]. Their distress can be attributed, directly or indirectly via emotional over-involvement, to anxiety for the patient [8, 9]. It is important to improve tendencies towards anxiety of family caregivers of young patients with psychotic disorders.

Family psychoeducation has been established as an evidence-based practice that primarily targets avoiding the relapse and rehospitalisation of patients with schizophrenia [10]. This intervention also consistently improves caregivers’ knowledge and self-efficacy, but whether the intervention has beneficial effects on their psychological wellbeing, care burden, or expressed emotion is as yet unclear [11]. In terms of emotional stress, involving anxiety, depression, or anger, a few studies have reported that psychoeducational interventions improved caregivers’ negative emotions, compared to control conditions [12, 13]. The trial conducted by Hazel et al. [8] showed that multiple-family group treatment reduced the integrated distress of caregivers, which was operationalised as the standardised and averaged outcomes of depression, anxiety, anger, and perceived stress. However, other randomised controlled trails have failed to demonstrate a significant difference between multi-family groups and control groups [14–17]. The primary outcome of many other studies was not the effect of family psychoeducation on caregivers’ emotional stress [18–20]. In addition to the inconsistency, few trials have focused on caregivers of young people with schizophrenia, who are more likely to be in earlier stages of psychosis and share similar problems relevant to the younger generation (e.g. work, marriage). A homogenous group intervention might be more effective to address the concern caregivers have about young patients with the mental disorder.

The Japanese Network of Psychoeducation and Family Support Program (JNPF) has developed a new approach, named SM-FPE [21]. This model focuses on the strength of caregivers, which is defined as the power a family has for dealing with difficulties when caring for people with severe mental disorders. In the intervention, the affirmation of caregivers’ coping behaviours is incorporated into problem-solving techniques; this design allows caregivers to reframe their viewpoints on problems, and helps them realise their inner strengths as well as alleviate their tendencies towards emotional stress. A quasi-randomised controlled trial found that SM-FPE lowered the relapse rate of schizophrenia [22]. However, there has been little evidence regarding the positive effects of SM-FPE on the mental health of people who are engaged in informal care of an individual with a mental disorder [23–25]. This study thus investigated whether SM-FPE plus TAU is more effective than TAU alone for reducing anxiety and other burdens in family caregivers of young patients with psychotic disorders.

## Methods

### Participants

Subjects in this study were patients with psychotic disorders and their primary caregivers. Inclusion criteria for patients were to be aged between 15 and 39 years, to currently receive outpatient treatment, and to meet the diagnostic criteria of the Diagnostic and Statistical Manual of Mental Disorders, Fourth Edition, Text Revision (DSM-IV-TR) for schizophrenia, brief psychotic disorder, schizophreniform disorder, schizoaffective disorder, or delusional disorder [26]. The diagnosis of the patients was confirmed by the study’s investigators according to the DSM-IV-TR. We excluded patients who were diagnosed with mental retardation or cluster B personality disorders by psychiatrists in charge.

Inclusion criteria for caregivers were to be aged between 20 and 74 years, to be classified as having one of the following relationships with a patient: parent, spouse, sibling, or to have been living with the patient for more than 3 months, and to play a primary role in the care of the patient. We excluded caregivers who had been judged not to be suitable for participating in this study for any reason by a psychiatrist in charge of the patients.

### Study design

This was a parallel-group study stratified by recent-onset/chronic psychosis and implementation site in Japan. The clinical stages of psychosis were distinguished as either ≤59 or ≥ 60 months after the onset of psychotic symptoms [27]. The study settings were four mental hospitals that covered the western side of the medical regions of a prefecture in the middle of Japan.

Randomisation of families to either a ‘SM-FPE plus TAU’ group or a ‘TAU alone’ group, stratified by time since the onset of psychotic symptoms and hospital, was performed by an independent statistician, according to a 1:1 allocation sequence [28]. Allocation was concealed from investigators enrolling and assessing participants, in that the statistician generated the sequence at his office and informed the investigators of the results only after enrolled participants had completed baseline assessments.

### Study setting

The programme was carried out at four mental hospitals: Yagoto Hospital, Minami-chita Hospital, Kusunoki Mental Hospital, and Toyota-nishi Hospital in Aichi Prefecture. Yagoto and Kusunoki Mental Hospital are in Nagoya, which is the third largest city in Japan. Toyota-nishi Hospital is in Toyota, which is a typical medium-size city in the country. Minami-chita Hospital covers the rural medical region of the Chita Peninsula.

### Interventions

SM-FPE was composed of an educational session (45 min), a break (15 min), and a group session (60 min), which took place every 2 weeks over a course of 8 weeks. The group-format programme was conducted by a multidisciplinary team of three to six members chosen from psychiatrists, clinical psychologists, nurses, occupational therapists, and psychiatric social workers. Individual groups included three to five primary caregivers, but not their ill relatives. The educational sessions were provided to a group of caregivers as interactive lectures adapted to the problems that young patients with psychosis often face. The content of the lectures covered diagnosis, prognosis, aetiology, symptoms, drug treatment, communication skills, and social resources. The information was based on the normalization approach, which regards psychotic symptoms as lying on a continuum of psychotic-like experiences that healthy individuals can experience [29]. A problem-solving approach was applied in the group sessions, but SM-FPE further focused on the strength of caregivers and did not include the patients.

Patients in both the intervention and the control groups received TAU. Their attending psychiatrists provided outpatient treatment, which primarily consisted of pharmacotherapy and supportive psychotherapy on a bi-weekly or 4-weekly basis. Case management, occupational therapy, or day-care programs were available depending on individual patient needs. If caregivers had been members of self-help groups before the allocation, they continued to attend their group sessions. Additionally, if caregivers used psychiatric services and/or psychotropic drugs before the allocation, they continued to use them.

### Training and fidelity assessment

All staff members completed a 2-day workshop that was approved by the JNPF. The first, third, and fourth authors are SM-FPE instructors certified by the same organisation, but they were assigned the same roles as all other staff in the programme. In order to confirm the fidelity of the programme implementation, all group sessions were recorded, and, after the completion of all sessions, 20% of them were randomly selected and assessed on a fidelity scale with eight items related to structured group work and eight items related to staff roles. A certified instructor evaluated the former items with two options (yes/no) and the later items with three grades (all play/some play/none play). This instrument was developed by the JNPF, but its validity and reliability have not been confirmed.

### Assessment measures

All outcome measures were assessed at baseline, post-intervention (10 weeks), and 1 month after the end of the intervention (14 weeks). The primary outcome was the trait anxiety of family caregivers at 14 weeks. The reason for the selection of the primary outcome was to investigate whether the strength-based intervention could modify the predisposition to anxiety behind state and psychosis-specific anxiety through changing how to perceive stressful situations. A previous study [30] suggested that a preliminary intervention had the potential to modify traits related to anxiety. Since a brief family intervention did not show long-term effects [15], the trait reduction was considered important because it could represent a persistent effect on caregivers’ responses to anxiety-provoking situations. The secondary outcomes for family caregivers were state anxiety, psychological distress, care burden, expressed emotion, and stigma. In addition to the caregivers’ outcomes, the patient’s overall level of functioning was evaluated as a secondary outcome. We also assessed the kinds of antipsychotic drugs prescribed to patients and the total antipsychotic dose converted to chlorpromazine equivalent [31].

#### State-trait anxiety inventory (STAI)

The STAI is a 40-item self-report questionnaire that measures trait and state anxiety. Trait anxiety (T-anxiety) is the extent to which an individual is predisposed to become anxious, whereas state anxiety (S-anxiety) is the severity of the anxiety experienced by an individual at a given time. Half of the items are reversed as measures of positive trait and state, which represents the absence of anxiety. Both types of anxiety are separately assessed and each total score ranges from 0 to 60. T-anxiety items include, ‘I worry too much over something that really doesn’t matter’ and ‘I am content’. S-anxiety items include, ‘I am presently worrying over possible misfortunes’ and ‘I feel content’ [32]. The reliability and validity of the Japanese version of this questionnaire have been confirmed [33].

#### K6

This is a short (six-item) self-report screening tool that was originally developed to detect depressive and anxiety disorders in the general population [34]. The total score ranges from 0 to 24 and a cut-off of 9 was adopted in a validation study in Japan. K6 items include, ‘How often do you feel so depressed that nothing could cheer you up?’ and ‘How often do you feel nervous?’ [35]. In addition to its use in screening, the K6 has been used as a measure of severity of psychological distress due to depression and anxiety [36].

#### Japanese version of the Zarit burden interview Short version (J-ZBI_8)

The Zarit Burden Interview is a 22-item self-report scale to assess the burden of care in the impaired elderly [37]. The burden is associated with caregiver’s depression and applicable to assess caregivers of patients with psychosis [38, 39]. The reliability and validity of the Japanese version have been confirmed [40]. Arai et al. developed the eight-item short version, with a total score ranging from 0 to 32. The J-ZBI_8 comprises two factors: ‘personal strain’ and ‘role strain’. The former includes items such as, ‘Do you feel embarrassed over your relative’s behaviour?’; the latter includes items such as, ‘Do you feel your social life has suffered because you are caring for your relative?’ [41, 42].

#### Family attitude scale (FAS)

The FAS is a 30-item self-report scale to assess expressed emotion (EE). This emotion can be characterised as the caregivers’ feelings of anger and anxiety regarding their state of hostility towards, criticism of, or over-involvement with patients with schizophrenia [9]. Each item score is summed up to provide a total score that ranges from 0 to 120. FAS items include, ‘He deliberately causes me problems’ and ‘I find myself saying nasty or sarcastic things to him’ [43]. A higher total score is significantly correlated with higher levels of criticism (r = 0.44) and hostility (r = 0.41) in the Camberwell Family Interview [44]. The Japanese version of the FAS has been validated [45].

#### Link’s stigma scale (LSS)

This self-report instrument with 12 questions is designed to measure the devaluation and discrimination that patients, family members, and general citizens perceive regarding mental illnesses. Each question score is summed up to provide a total score that ranges from 0 to 36. LSS items include, ‘Most people think less of a person who has been in a mental hospital’ and ‘Most people in my community would threat a former mental patient just as they would treat anyone’ [46]. The reliability and validity of the Japanese version have been confirmed [47].

#### Global assessment of functioning (GAF)

This scale is used to report patients’ overall functioning. The GAF is scored from 0 to 100 with respect to psychological, social, and occupational functioning [26]. High scores on the GAF correspond to better functioning. Psychiatrists in charge evaluated the patients’ level of functioning and, to ensure blinding, participants were asked not to inform the doctors of assignment results.

### Sample size

We referred to a previous single-arm study that explored the effects of a psychoeducational intervention on T-anxiety of 46 relatives of patients with schizophrenia [30]. The study results indicated that each group needed a sample size of 35 participants, assuming a 10% dropout rate, to detect a reduction of 6 points (SD = 7.2) in the total T-anxiety score at 14 weeks with a two-tailed significance level of 5% and a power of 90%.

### Statistical analysis

All analyses were conducted in accordance with the intent-to-treat (ITT) model. If there were no missing values, analysis of covariance (ANCOVA) was used to examine group effects after adjusting for the baseline scores. If missing values were observed, we used linear mixed models. Subsequently, pre-specified subgroup analyses were conducted to investigate the differences between recent-onset and chronic groups.

In post-hoc analyses, structural equation modelling (SEM) was used to examine group effects on the overall state of caregivers’ mental health. An exploratory factor analysis of S-anxiety, K6, J-ZBI_8, FAS, and LSS at baseline was carried out, using the principal factor method, with eigenvalues > 1 being used as the criterion to select the number of factors. Variables with low communality were dropped to determine the components of the poor mental health state. Means and SDs of all variables used in the SEM were calculated and were correlated with each other. In the SEM, we created a path model of the conceptual framework of the caregivers’ mental health state according to the flow of this randomised controlled trial (RCT). The fit of the model to the data was computed in terms of a chi-squared (CMIN), comparative fit index (CFI) and the root mean square error of approximation (RMSEA). According to conventional criteria, a good fit would be indicated by CMIN/*df* < 2, CFI > 0.97, and RMSEA < 0.05, and an acceptable fit by CMIN/*df* < 3, CFI > 0.95, and RMSEA < 0.08 [48]. Since we were specifically interested in young people with schizophrenia in earlier stages of psychosis, a multiple-group analysis was performed to explore the differences in the intervention effects between recent-onset and chronic psychoses. Beginning with a non-constrained model, we compared a more constrained model with a less constrained model. The null hypothesis was defined as the model with less constrains being correct. If the χ^2^-values of the two models did not differ at a statistically significant level, we assumed that the model with more constrains was correct. A significant difference between the two stages of schizophrenia was indicated when the *z*-value of a paired comparison was the critical ratio of > 1.96.

A two-tailed *p-*value of < 0.05 was set to test the null hypotheses. All statistical analyses were calculated using PASW Statistics version 20 and Amos version 20 for Windows (IBM Software Japan, Tokyo, Japan). The statistician who performed the analyses was blinded to the allocation in the study.

## Results

### Enrolment and baseline characteristics of the participants

**Fig. 1:**
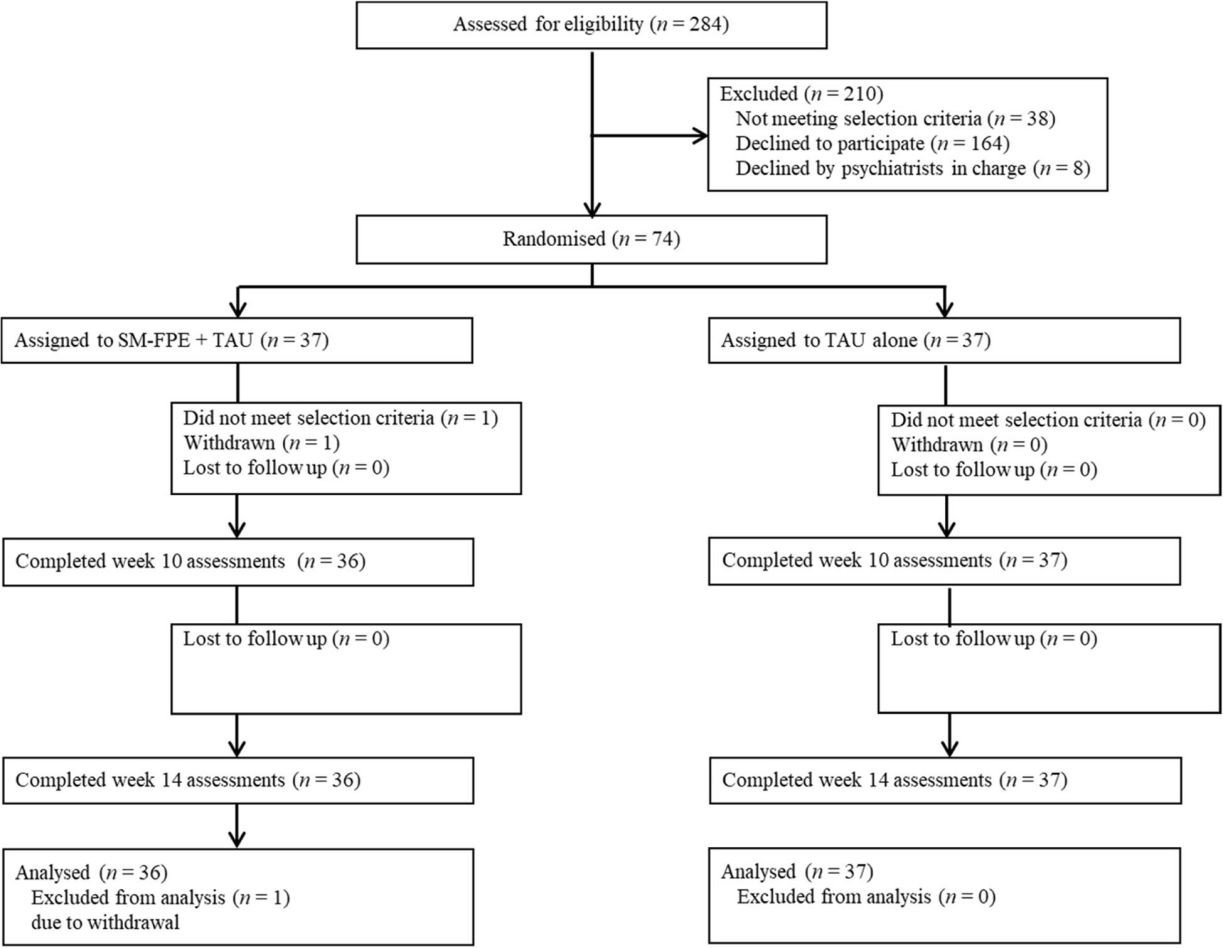
Participant flow diagram

The trial started in July 2012 and ended in January 2016. Fig. 1 shows the study flow; we screened 284 family caregivers, 238 of whom met the eligibility criteria. Out of 74 participants, 37 were randomly assigned to receive SM-FPE plus TAU and 37 to receive TAU alone. The number of participants at each facility was 24 (Yagoto Hospital), 21 (Kusunoki Mental Hospital), 12 (Toyota-nishi Hospital), and 17 (Minami-chita Hospital). A protocol deviation occurred for one participant in the intervention group. We noticed after randomisation that her daughter still remained hospitalised. We estimated however that this violation had only a very small influence on the outcomes, because she was discharged on the day of the first session. Table 1 shows sociodemographic and clinical characteristics at baseline. Clinical characteristics were similar between the assigned arms. Most caregivers were female (82.2%), and predominantly mothers (96.7%). Almost all patients were diagnosed with schizophrenia (97.3%), about one third in the recent-onset condition (35.6%). Although the ratio of female to male was 1:1.9 and that of compulsory to post-compulsory education was 1:2.8, most patients were unemployed (76.7%), unmarried (95.9%), and dependent on the caregivers for their lives (90.4%).

**Table 1:** Sociodemographic and clinical characteristics of the participants

Characteristics (caregivers)	SM-FPE + TAU		TAU alone		All participants	
	(*n* = 36)		(*n* = 37)		(*n* = 73)	
Age, mean (SD), years	58.9	(6.4)	58.2	(9.4)	58.4	(8.0)
Sex, n (%)						
Female	30	(83.3)	30	(81.1)	60	(82.2)
Male	6	(16.7)	7	(18.9)	13	(17.8)
Relationship, n (%)						
Mother	30	(83.3)	29	(78.4)	58	(80.8)
Father	6	(16.7)	6	(16.2)	12	(16.4)
Spouse	0		1	(2.7)	1	(1.4)
Sibling	0		1	(2.7)	1	(1.4)
Education, n (%)						
< High school	0		1	(2.7)	1	(1.4)
High school	22	(61.1)	16	(43.2)	38	(52.1)
Two-year college	10	(27.8)	12	(32.4)	22	(30.1)
University	4	(11.1)	8	(21.6)	12	(16.4)
Occupation, n (%)						
Employed, full-time	12	(33.3)	15	(40.5)	27	(37.0)
Employed, part-time	8	(22.2)	9	(24.3)	17	(23.3)
Homemaker	12	(33.3)	8	(21.6)	20	(27.4)
Retirement	4	(11.1)	5	(13.5)	9	(12.3)
Marital status, n (%)						
Unmarried	1	(2.8)	1	(2.7)	2	(2.7)
Married	29	(80.6)	28	(75.7)	57	(78.1)
Divorced	3	(8.3)	6	(16.2)	9	(12.3)
Widowed	3	(8.3)	2	(5.4)	5	(6.8)
Psychotropic use/psychiatrist’s visit, n (%)	4	(11.1)	7	(18.9)	11	(15.1)
Self-help group, n (%)	6	(16.7)	6	(16.2)	12	(16.4)
K6 ≥ 9, n (%)	13	(36.1)	14	(37.8)	27	(37.0)
Age, mean (SD), years	29.7	(5.6)	30.5	(5.7)	30.1	(5.6)
Sex, n (%)						
Female	11	(30.6)	14	(37.8)	25	(34.2)
Male	25	(69.4)	23	(62.2)	48	(65.8)
Education, n (%)						
< High school	8	(22.2)	11	(29.7)	19	(26.0)
High school	12	(33.3)	12	(32.4)	24	(32.9)
Two-year college	6	(16.7)	6	(16.2)	12	(16.5)
University	10	(27.8)	8	(21.6)	18	(24.7)
Occupation, n (%)						
Unemployed	27	(75.0)	29	(78.4)	56	(76.7)
Employed, full-time	2	(5.6)	2	(5.4)	4	(5.5)
Employed, part-time	4	(11.1)	4	(10.8)	8	(11.0)
Homemaker	1	(2.8)	1	(2.7)	2	(2.7)
Sheltered work	2	(5.6)	1	(2.7)	3	(4.1)
Marital status, n (%)						
Unmarried	34	(94.4)	36	(97.3)	70	(95.9)
Married	1	(2.8)	1	(2.7)	2	(2.7)
Divorced	1	(2.8)	0		1	(1.4)
Living status, n (%)						
Living alone	0		3	(8.1)	3	(4.1)
Living with a participating caregiver	33	(91.7)	33	(89.2)	66	(90.4)
Living with someone not engaging in care	2	(5.6)	1	(2.7)	3	(4.1)
Group home	1	(2.8)	0		1	(1.4)
Diagnosis, n (%)						
Schizophrenia	35	(97.2)	36	(97.3)	71	(97.3)
Brief psychotic disorder	1	(2.8)	0		1	(1.4)
Schizoaffective disorder	0		1	(2.7)	1	(1.4)
Duration of the disorders						
Mean (SD), months	86.6	(67.1)	101	(69.5)	93.9	(68.2)
< 59 months	13	(36.1)	13	(35.1)	26	(35.6)
≥ 60 months	23	(63.9)	24	(64.9)	47	(64.4)
Number of hospitalisations, mean (SD)	1.7	(1.2)	1.8	(1.7)	1.8	(1.5)

### Attrition and study integrity

#### Attrition

One mother withdrew her consent to participate in this trial just after the randomisation. Thus, data from 73 participants were available for ITT analyses. All 36 participants in the SM-FPE plus TAU group completed the intervention, and they attended a mean number of 4.8 of five sessions (SD = 0.49). No data were missing for the participants who were included in the analyses. In the TAU alone group, one participant started a new psychotropic/psychiatric therapy and two participants entered new self-help groups during the study period.

#### Fidelity assessment

Ten randomly selected sessions satisfied 100% of eight items for the structured group work and 81% of eight items for the roles of staff (all play/some play). Both percentages demonstrate that the implementation adequately adhered to the standard model of the JNPF.

#### GAF assessment

The κ-values for agreement between the allocation states and those speculated by psychiatrists in charge were 0.23 (95% CI: 0.01–0.46) at 10 weeks and 0.18 (95% CI: − 0.04–0.41) at 14 weeks. Both values suggest that the blinding level of the assessments was acceptable.

#### Patient hospitalisation

The numbers of patients who were admitted during the study period were one and three in the intervention and control group, respectively.

#### Primary and other outcomes

ANCOVA was conducted on T-anxiety of family caregivers (Table 2). No statistically significant differences were detected between the two groups at week 10 (*p* = 0.19) and week 14 (*p* = 0.24), after adjusting for group differences in baseline scores. ANCOVA was also used to analyse secondary outcomes (Table 2). The analyses did not detect statistically significant differences between groups in S-anxiety, K6, J-ZBI_8, FAS, LSS, GAF, or the kinds and amounts of antipsychotics at the two assessment points, after adjusting for group differences in respective baseline scores.

**Table 2:** Adjusted results for the outcomes of the participants, mean (SE), ANCOVA

	Baseline				10 Weeks						14 Weeks					
Measure	SM-FPE + TAU		TAU alone		SM-FPE + TAU		TAU alone		*F*	*p*	SM-FPE + TAU		TAU alone		*F*	*p*
Caregivers																
Trait anxiety	46.6	(1.7)	49.4	(1.9)	42.9	(1.5)	46.0	(1.7)	1.73	0.19	41.6	(1.6)	44.2	(1.6)	1.68	0.20
State anxiety	48.5	(1.8)	49.0	(1.5)	41.0	(1.8)	46.4	(1.7)	1.84	0.18	41.7	(1.7)	45.2	(1.8)	2.06	0.16
K6	6.8	(0.8)	7.2	(0.9)	4.9	(0.7)	5.1	(0.7)	0.11	0.74	4.3	(0.6)	5.7	(0.7)	0.58	0.45
J-ZBI_8	12.3	(1.3)	10.9	(1.2)	9.3	(1.1)	10.9	(1.2)	< 0.01	0.94	9.1	(1.1)	9.1	(1.1)	0.01	0.95
FAS	46.8	(4.2)	46.9	(3.5)	36.6	(3.6)	45.4	(3.7)	0.75	0.39	37.8	(3.9)	42.2	(3.4)	0.79	0.38
LSS	35.7	(6.0)	35.7	(0.8)	34.0	(0.9)	35.9	(1.0)	0.57	0.45	34.4	(1.1)	35.2	(1.0)	0.50	0.48
Patients																
GAF	46.6	(2.4)	48.5	(1.9)	47.5	(2.3)	50.1	(2.2)	0.60	0.44	47.1	(2.5)	50.6	(2.4)	0.77	0.38
Kinds of APs	1.8	(0.2)	1.9	(0.2)	1.8	(0.2)	1.8	(0.2)	0.14	0.71	1.8	(0.2)	1.8	(0.2)	0.11	0.75
Amount of APs, mg	810	(112)	780	(96)	806	(116)	760	(95)	0.07	0.80	795	(116)	744	(94)	0.08	0.77

**Fig. 2:**
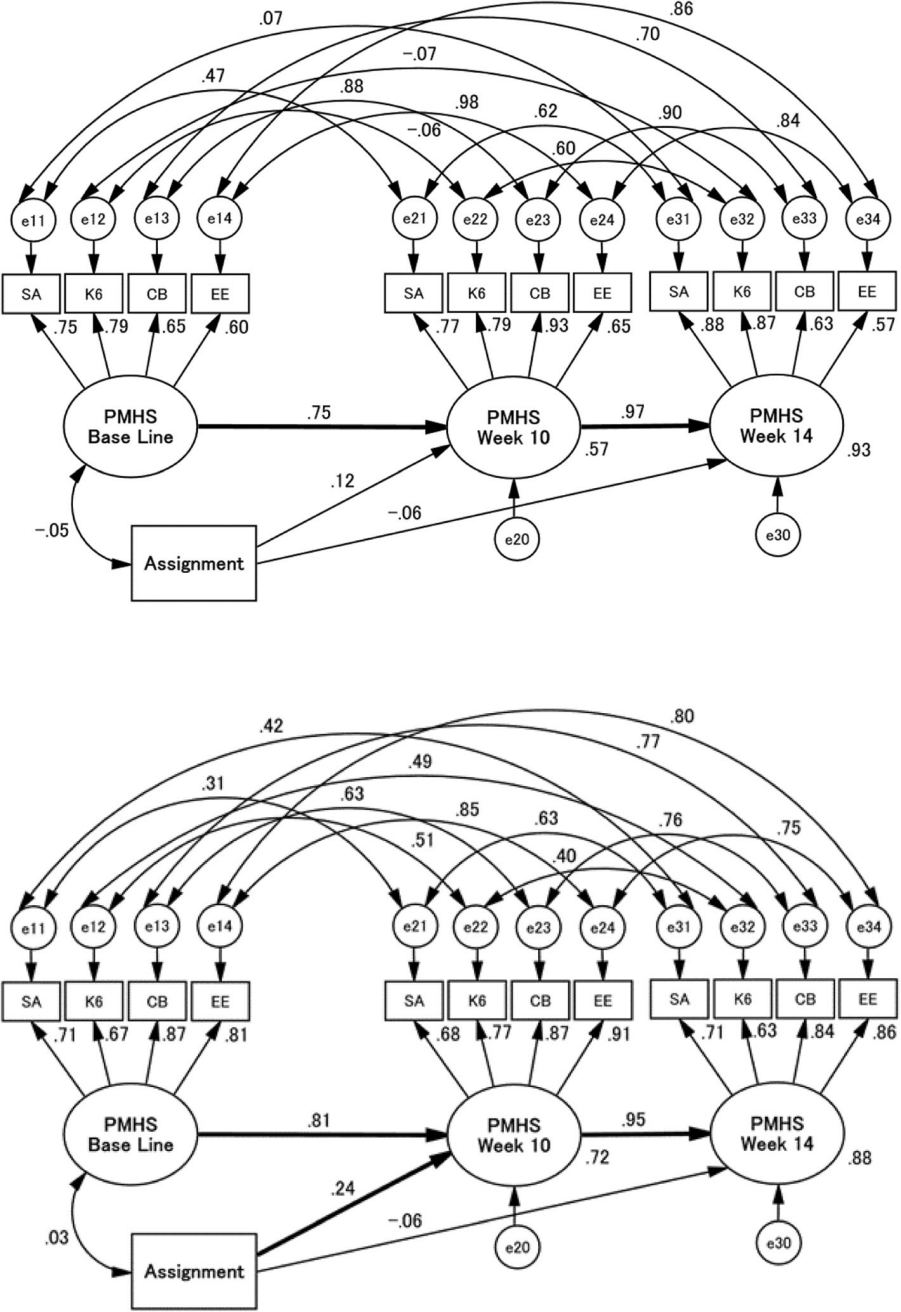
Path model of the stratification of recent-onset psychosis (right) and chronic psychosis (left) according to the study flow

In the post-hoc analyses, the principle factor method found a single factor with an eigenvalue > 1; the scree plot also indicated the presence of a single factor. We excluded LSS from the measurement model of the overall state of caregivers’ mental health, because the communality of LSS (0.039) was clearly lower than that of S-anxiety, K6, J-ZBI_8, or FAS (0.649, 0.716, 0.602, or 0.481, respectively). Variables were more significantly correlated with each other except for assignment in chronic psychosis than in recent-onset psychosis (Table 3). The path model provided acceptable fit to the data: CMIN/*df* = 1.671, CFI = 0.962, and RMSEA = 0.097. However, when the results of the recent-onset and chronic groups were compared in the original model, the chi-square difference between the non-constrained and measurement models reached a statistically significant level of 0.05 (*p* = 0.025). This suggested that both groups could be regarded as different in the above model, which provided acceptable fit to the data: CMIN/df = 1.496, CFI = 0.945, and RMSEA = 0.084. Fig. 2 illustrates that the intervention effects were not significant at 10 and 14 weeks in the recent-onset stage (*p* = 0.429 and 0.445, respectively), while in the chronic stage, they were significant at the end of the programme (*p* = 0.012) but not at a 1-month follow-up (*p* = 0.361). Between the two stages, the paired comparisons of both intervention effects at 10 and 14 weeks did not reach the level of statistical significance (*z* = 0.46 and 0.20, respectively).

**Table 3:** Correlations among variables used in structural equation modelling

	1	2	3	4	5	6	7	8	9	10	11	12	13
1. Assignment (1, SM-FPE + TAU, 2 TAU alone)	–	−.21	.00	−.21	.08	.13	.10	.00	.15	.01	.17	−.11	.04
2. State anxiety, base line	.15	–	.45*	.60**	.56**	.69***	.57**	.71***	.57**	.66***	.57**	.68***	.55**
3. K6, base line	.08	.58**	–	.61**	.64***	.28	.38	.52**	.50*	.52**	.43*	.51**	.62**
4. J-ZBI, base line	−.01	.61***	.61***	–	.58**	.45*	.47*	.72***	.49*	.44*	.35	.83***	.57**
5. FAS, base line	−.05	.50***	.48**	.74***	–	.25	.30	.51**	.89***	.26	.41*	.67***	.88***
6. State anxiety, week 10	.35*	.55***	.41**	.40**	.45**	–	.78***	.77***	.45*	.84***	.74***	.57**	.36
7. K6, week 10	−.02	.54***	.71***	.54***	.60***	.52***	–	.78***	.48*	.76***	.86***	.59**	.37
8. J-ZBI_8, week 10	.18	.54***	.55***	.76***	.60***	.58***	.69***	–	.69***	.77***	.77***	.83***	.61**
9. FAS, week 10	.22	.51***	.45**	.63***	.81***	.63***	.73***	.79***	–	.39*	.60**	.65***	.87**
10. State anxiety, week 14	.29*	.57***	.48**	.38**	.44**	.79***	.57***	.55***	.61***	–	.81**	.52**	.40*
11. K6, week 14	.21	.47**	.61***	.34*	.39**	.62***	.68***	.52***	.54***	.68***	–	.56**	.49*
12. J-ZBI_8, week 14	.06	.48**	.49***	.78***	.64***	.50***	.61***	.89***	.74***	.54***	.50***	–	.71***
13. FAS, week 14	.14	.34*	.38**	.54***	.78***	.55***	.65***	.70***	.89***	.60***	.50***	.76***	–
Mean	1.5	52.0	8.2	12.4	46.7	47.4	6.2	10.8	42.3	49.4	6.8	9.7	43.6
	1.5	46.9	6.3	11.1	47.0	41.7	4.3	9.7	40.3	40.2	4.0	8.8	38.0
SD	0.5	9.3	5.3	8.3	25.2	11.3	4.3	7.1	22.8	10.6	4.5	6.3	23.5
	0.5	9.8	4.8	7.0	22.3	10.0	3.6	7.0	22.6	9.4	3.3	6.6	21.0

Upper figure reflects recent-onset psychosis (*n* = 26), lower figure reflects chronic psychosis (*n* = 47): *p < .05; **p < .01; ***p < .001

## Discussion

We failed to demonstrate effects of SM-FPE on T-anxiety and other individual outcomes of family caregivers of young adults with schizophrenia. Our analysis indicates that our failure to prove the usefulness of the family intervention can be partly attributed to a lack of effectiveness in the integrated outcome of the caregivers of the recent-onset patients, in which the factor loadings of anxiety and depression were higher than those of care burden and expressed emotion.

A main reason for these negative results may be the use of general measures to assess negative emotions. Although our primary interest was the caregivers’ predisposition to anxiety, a psychosis-specific questionnaire might be more sensitive to evaluate their state anxiety (e.g. the Involvement Evaluation Questionnaire) [49]. Another reason may be floor effects in the assessment measures. In the case of the STAI, scores of 20.5% for T-anxiety and 30.1% for S-anxiety at baseline were less than 30% of each standard score, corresponding to the category of low anxiety [50]. Other possible reasons are the natural course of anxiety and social desirability biases, which can be seen in the decrease of 5.2 points in T-anxiety and that of 7.3 points in S-anxiety in the TAU alone group, from baseline to 14 weeks, although the patients’ condition had undergone very little change. These types of reductions could enhance floor effects on the outcomes. Despite these factors that might undermine the evaluation of the usefulness of the intervention, this pragmatic study did not exclude caregivers with subthreshold anxieties, because such selection does not usually occur in a clinical setting. Furthermore, the SM-FPE has been considered incapable of improving caregivers’ emotional distress [25]. The multiple-group analysis suggests that the standard intervention needs to be improved to alleviate the anxiety and depression of caregivers of young people with recent-onset psychosis more effectively.

The internal validity of this RCT was supported by low attrition and good adherence. According to a review of psychoeducational studies for families of people with schizophrenia, 17% of studies presented recruitment, retention, and engagement problems, and 38% offered no explicit data on these matters [11]. In our study, we observed almost no attrition from the randomisation to the post-intervention follow-up. Furthermore, almost all participants adhered to the programme. This implies that they considered it meaningful to continue with it, although we did not assess perceived usefulness of the programme through qualitative interviews with the participants. In the lecture component, caregivers could accept psychotic symptoms more easily by using the normalisation. The approach explained that people could hear hallucinations in normal situations, such as the voices that stranded climbers experienced in winter mountains [29]. In the group format component, the discussed problems were categorised into four themes: ‘how to deal with the symptoms and treatment of the illness’, ‘concerns about patients’ present and future lives’, ‘how to face communication with the patients’, and ‘how to support patients’ social engagement’. In terms of external validity, the trial was carried out in four psychiatric hospitals, which are located in the metropolis, a provincial city, and a rural area in central Japan. The multi-site implementation thus increased the representativeness of the study population and demonstrated that a multi-family group intervention could be integrated into routine practices and delivered by staff in mental hospitals across Japan.

The present study has a number of limitations. First, the characteristics of participants might differ from those of non-participants. According to the protocol approved by the Institutional Review Board and Ethics Committee, we had to guarantee the right that screened people could cease participation without reason. The rate of refusal in the screening (about 58%) might reduce the external validity of this study. Second, participants and researchers were aware of the allocation arm due to the nature of the RCT for psychological interventions. Lack of blinding might influence outcome assessments [51]. We had an independent data analyst to reduce detection bias, and patient outcome assessors were blinded to the allocation. Third, the participants were followed up for only 4 weeks from the end of the intervention. In chronic psychosis, the direct effects of SM-FPE on the overall state of caregivers’ mental health decreased during this short period. This suggests that booster sessions are needed to maintain the improvements. Fourth, the positive effects were revealed only by the post-hoc analyses that integrated different concepts in caregiving. However, the reason for the use of the SEM model was that it was considered to be a close representation of the clinical responses of the caregivers observed in the intervention. In further research, an a priori defined conceptual framework is needed to evaluate caregivers’ mental health in family interventions more robustly.

## Conclusions

The implementation of the SM-FPE did not modify caregivers’ T-anxiety or improve other individual outcomes. When their burdens were considered overall, there was evidence of an intervention benefit on the overall mental health state of the caregivers of the chronic patients. However, such intervention effects were not observed in the integrated outcome of those of the recent-onset patients. The intervention programme requires stronger evidence that it supports families before wider dissemination.

## List of abbreviations

ANCOVA: Analysis of covariance
AP: Antipsychotic
CB: Care Burden
CFI: Comparative fit index
CMIN: Chi-squared index
DSM-IV-TR: Diagnostic and Statistical Manual of Mental Disorders, Fourth Edition, Text Revision
EE: Expressed emotion
FAS: Family Attitude Scale
GAF: Global Assessment of Functioning
ITT: Intent-to-treat
JNPF: Japanese Network of Psychoeducation and Family Support Program
J-ZBI_8: Japanese version of the Zarit Burden Interview Short Version
LSS: Link’s Stigma Scale
PMHS: Poor Mental Health State
RCT: Randomised controlled trial
RMSEA: Root mean square error of approximation
SA: State anxiety
S-anxiety: State anxiety
SEM: Structural equation modelling
SM-FPE: Standard model of family psychoeducation
STAI: State-Trait Anxiety Inventory
T-anxiety: Trait anxiety
TAU: Treatment as usual
